# The Validity and Reliability of the Short Form of the Five Facet Mindfulness Questionnaire in Japan

**DOI:** 10.3389/fpsyg.2022.833381

**Published:** 2022-04-14

**Authors:** Toru Takahashi, Junichi Saito, Masahiro Fujino, Masashi Sato, Hiroaki Kumano

**Affiliations:** ^1^Faculty of Human Sciences, Waseda University, Tokorozawa, Japan; ^2^Comprehensive Research Organization, Waseda University, Tokyo, Japan; ^3^Human Information Science Laboratory, NTT Communication Science Laboratories, Atsugi, Japan; ^4^Open Innovation Institute, Kyoto University, Kyoto, Japan; ^5^Graduate School of Human Sciences, Waseda University, Tokorozawa, Japan

**Keywords:** mindfulness, five facet mindfulness questionnaire, short form, psychometrics, method effects, wording effects

## Abstract

**Background:**

A brief measure of dispositional mindfulness is important for applied research on mindfulness. Although short forms of the Five Facet Mindfulness Questionnaire (FFMQ), which measures the five aspects of mindfulness (i.e., observing, describing, acting with awareness, non-judging, and non-reactivity), have been developed worldwide, the validity and reliability of the Japanese version has not been examined. This study aims to examine the validity and reliability of the 24-item and 15-item versions of the FFMQ in Japan, which are the most widely used versions worldwide.

**Methods:**

Online surveys were conducted for 889 adults in Japan through an online survey company using self-reported questionnaires including the FFMQ to confirm the factor structure and validity. To examine construct validity, we examined the relationship between the short form of FFMQ and mind wandering, interoceptive awareness, experiential avoidance, cognitive fusion, openness, neuroticism, self-compassion, depression, and anxiety, which have been theoretically or empirically shown to be related to mindfulness. In addition, 137 adults responded to the FFMQ again, after four weeks, for the test-retest reliability.

**Results:**

The correlated five-factor and four-factor (excluding observing) models and the higher-order factor hierarchical model did not show sufficient goodness of fit, while the 24-item version showed acceptable fit when uncorrelated method factors loaded on by the positive and negative (reverse-scored) items were added. However, the 15-item version did not show acceptable fits for any of the models. Regarding reliability, the 24-item version showed acceptable values. In terms of the relationship between the original and the shortened version of the FFMQ, the 24-item version shared approximately 80% of the variance with the original one. In addition, although the wording effects of positive and negative items seemed to affect the correlations between the FFMQ and the other scales, the associations with related concepts were as predicted generally, supporting the construct validity of the short form of the FFMQ.

**Conclusion:**

In Japan, the 24-item version of FFMQ showed acceptable validity and reliability similar to the original version, and we recommend that the 24-item version be used.

## Introduction

Significant attention has been paid to mindfulness in the field of psychology and related disciplines. The most famous definition of mindfulness is “the awareness that emerges through paying attention on purpose, in the present moment, and non-judgmentally to the unfolding of experience moment by moment” by Kabat-Zinn (2003, p. 145), who developed the first standardized intervention, the mindfulness-based stress reduction program (Kabat-Zinn, 1982). The tendency of being mindful, or possessing dispositional mindfulness, has been linked to high well-being (Brown and Ryan, 2003; Iani et al., 2017). Furthermore, dispositional mindfulness has also been found to be a key mediating variable explaining the effects of mindfulness interventions on mental health problems such as depression and anxiety (Gu et al., 2015; Alsubaie et al., 2017). As mindfulness has become a promising concept in the field of practical or clinical psychology, its brief measurement is critical for investigating the relationship between dispositional mindfulness and various psychological concepts.

### Questionnaires of Dispositional Mindfulness

Most studies measured dispositional mindfulness by self-reported questionnaires. One of the most used questionnaires is the Five Facet Mindfulness Questionnaire (FFMQ; Baer et al., 2006), which was developed by conducting conjoint factor analysis for items included in the existing mindfulness questionnaires such as the Mindful Attention Awareness Scale (MAAS; Brown and Ryan, 2003). MAAS is also one of the most used questionnaires, which regards mindfulness as a single factor reflecting a tendency of being attentive to and aware of what is taking place in the present. In contrast, the FFMQ has been used to clarify more detailed mechanisms of mindfulness because it can measure mindfulness in terms of five aspects. The five aspects are observing, describing, acting with awareness, non-judging of inner experience, and non-reactivity to inner experience (Baer et al., 2006). The subscales that measure each aspect are described as follows (Baer et al., 2008; Bohlmeijer et al., 2011): Observing refers to noticing or attending to internal and external experiences, such as sensations, sights, and sounds. Describing refers to labeling internal experiences with words. Acting with awareness refers to attending to one’s activities of the moment (opposite of acting on automatic pilot). Non-judging of inner experience refers to taking a non-evaluative stance toward thoughts and feelings. Non-reactivity to inner experience refers to allowing thoughts and feelings to come and go without getting caught up in or carried away by them. A recent theory explaining the action mechanisms of mindfulness (Lindsay and Creswell, 2017) states that mindfulness is a combination of two elements, attention monitoring and acceptance, and that each of these elements can be measured separately with the FFMQ. (i.e., observing measures attention monitoring, while non-reactivity and non-judging measure acceptance). In addition, it has been shown empirically that each factor of the FFMQ is differently associated with different aspects of psychological well-being (Iani et al., 2017), therefore, measuring multiple aspects of mindfulness is important for practical applications.

### Short Versions of the Five Facet Mindfulness Questionnaire

Although the FFMQ has been translated and used frequently around the world, it has a relatively large number of items, 39 in total, which imposes some burden on the respondents. Therefore, some short versions of FFMQ have been developed to reduce the burden of responders. The short version of FFMQ given by Bohlmeijer et al. (2011) is one of the oldest and most used versions, which comprises 24 items. Bohlmeijer et al. (2011) developed the FFMQ by deleting 15 items from the perspective of item-total correlation, factor loadings, and redundancy of item content (correlated error terms). Although it is a shortened version created in Netherlands (Bohlmeijer et al., 2011), it is commonly used in the English-speaking world (e.g., Pelham et al., 2019). As another typical shortened version, Baer et al. (2012) created a 15-item version by selecting three items for each facet based on the item contents and the factor loadings calculated by factor analysis in Baer et al.’s (2006) study. Subsequently, Gu et al. (2016) showed convergent validity of the 15-item version in relation to depression and negative rumination, and acceptable internal consistency. Although several other shortened versions have been developed in different countries (e.g., Tran et al., 2013; Meng et al., 2020), these two versions are the most commonly used with their psychometric properties well examined (e.g., Medvedev et al., 2018; Pelham et al., 2019). However, the validity and reliability of the Japanese versions of these FFMQ shortened versions have not yet been examined. As more and more interventions using mindfulness are being conducted in Japan (Sado et al., 2018; Takahashi et al., 2019, 2020b), a self-assessment scale for mindfulness that is easy to use and less burdensome, is necessary.

### Aims and Hypotheses

Therefore, we aimed to develop the Japanese short form of FFMQ by examining the validity and reliability of the short forms developed by Bohlmeijer et al. (2011) and Baer et al. (2012). First, we examine whether the factor structure that has been shown to have a good fit in the original FFMQ can also be shown in the shortened version. Early studies of the FFMQ have shown that a model in which five factors are correlated with each other and a hierarchical model that assumes a second-order factor loaded on by the five factors provide a good fit (Baer et al., 2006; Sugiura et al., 2012). In addition, in the general population not selected in terms of mindfulness meditation experience, the four facet correlated factors model and the hierarchical model, excluding the observing facet, have also shown high goodness of fit (Baer et al., 2006; Sugiura et al., 2012; Gu et al., 2016). Contrarily, in recent years, it has been shown that models that add method factors loaded on by the positive and negative (reverse-scored) items, respectively, have an even higher goodness of fit (Van Dam et al., 2012; Lecuona et al., 2020). Particularly in Japan, there have been some instances where a psychological scale with a single factor in Western countries, has been divided into positive and negative item factors in the Japanese version (e.g., Hidano et al., 2000). Additionally, there was a case where the goodness of fit was improved by including a method factor loaded on by the negative items (e.g., Himichi et al., 2017). Therefore, we examine whether adding method factors to the model of the FFMQ improves the goodness of fit and explore a reasonable factor structure.

Second, this study examines the reliability of the shortened versions of the FFMQ. The original Japanese version of the FFMQ shows sufficient internal consistency (Cronbach’s alpha > 0.7), except for non-reactivity (Sugiura et al., 2012). In contrast, the test-retest reliability of the shortened versions of the FFMQ has not been examined in Japan. We expect that the short forms of the FFMQ will show sufficient test-retest reliability given that they are considered to measure a relatively stable tendency of mindfulness.

Finally, we confirm the validity of the short forms of FFMQ. As a first, we expect that the subscales of the shortened FFMQ will show very high correlations (i.e., *r* values ≥ 0.9) with the same subscales of the original FFMQ, which means that the shortened and original versions of the FFMQ have the almost same information. Next, to examine the construct validity of the shortened version, we examine whether the correlations with concepts that can be assumed to be associated with mindfulness, will be found in the shortened version. The hypotheses described below are summarized in Table 1. The acting with awareness subscale of the FFMQ includes some items from the MAAS, so it is expected that the acting with awareness in the short version of the FFMQ correlates strongly (*r* > 0.7) with the MAAS. The MAAS has also been shown to have a moderate negative correlation with the mind-wandering tendency (Kajimura and Nomura, 2016), thus it is expected that acting with awareness also has a moderate negative correlation (–0.7 < *r* < –0.4) with the mind-wandering tendency. In addition, mindfulness practice emphasizes awareness of interoceptive sensations such as bodily sensations, and in fact, each facet of the FFMQ has shown small to moderate positive correlations with each subscale of the Multidimensional Assessment of Interoceptive Awareness (MAIA; Shoji et al., 2018). Therefore, we expect similar correlations to be found between the shortened versions of the FFMQ and MAIA. As with the original version of the FFMQ given by Sugiura et al. (2012), the facets excluding observing in the short version are expected to show small negative correlations (–0.4 < *r* < –0.2) with experiential avoidance, which is described as “the attempt to escape or avoid private events, even when the attempt to do so causes psychological harm” (Hayes, 2004, pp. 649–650). The reason that observing does not show an adaptive association is that the general population that is not selected in terms of meditation experiences is not necessarily capable of non-judgmental and unbiased observation (Baer et al., 2008; Sugiura et al., 2012). Experiential avoidance is a major intervention target of Acceptance and Commitment Therapy (ACT; Hayes, 2004), one of the psychotherapies that use mindfulness. Another major process targeted by mindfulness in the ACT is cognitive fusion. Cognitive fusion is described as “the tendency for behavior to be overly regulated and influenced by cognition” (Gillanders et al., 2014, p. 84). In terms of decentering with thoughts, it is expected to show moderate negative correlations between non-judging and non-reactivity on the FFMQ and cognitive fusion. The relationship between dispositional mindfulness and big five personality has also been examined to test the discriminant and convergent validity of mindfulness measures (Baer et al., 2006; Bohlmeijer et al., 2011). We expect that small to moderate positive correlations will be found between openness and observing and describing in the short form of the FFMQ, as shown in Bohlmeijer et al. (2011). We also expect that small to moderate negative correlations will be found between neuroticism and facets, except for observing, as shown in Bohlmeijer et al. (2011). In addition, in mindfulness training, it has been established that a close relationship exists between mindfulness and self-compassion (Kuyken et al., 2010; Feldman and Kuyken, 2011) which is described as “being touched by and open to one’s own suffering, not avoiding or disconnecting from it, generating the desire to alleviate one’s suffering and to heal oneself with kindness” (Neff, 2003, p. 87). Further, small to moderate positive correlations have been shown between facets of the FFMQ and self-compassion (Baer et al., 2006), thus we expect similar small to moderate positive associations with the shortened Japanese version. In terms of clinical outcome, mindfulness interventions have been shown to have the most robust effect on internalizing symptoms such as depression and anxiety (e.g., Strauss et al., 2014). In terms of individual differences in dispositional mindfulness in the general population, the facets excluding observing have shown small to moderate negative correlations with depression and anxiety (Sugiura et al., 2012; Takahashi et al., 2020a), thus suggesting that similar correlations will be found with the short forms of the FFMQ in Japan. Based on the above expectation, the construct validity of the shortened version of the FFMQ will be examined.

**TABLE 1:** Hypotheses about Pearson’s correlations between the subscales of the short form of the five facet mindfulness questionnaire (FFMQ) and the related concepts.

	Observing	Describing	Acting with awareness	Non-judging	Non-reactivity
Mindful attention and awareness			*r* > 0.7		
Mind-wandering			–0.7 < *r* < –0.4		
Interoceptive awareness	0.2 < *r* < 0.7	0.2 < *r* < 0.7	0.2 < *r* < 0.7	0.2 < *r* < 0.7	0.2 < *r* < 0.7
Experiential avoidance		–0.4 < *r* < –0.2	–0.4 < *r* < –0.2	–0.4 < *r* < –0.2	–0.4 < *r* < –0.2
Cognitive fusion				–0.7 < *r* < –0.4	–0.7 < *r* < –0.4
Openness	0.2 < *r* < 0.7	0.2 < *r* < 0.7			
Neuroticism		–0.7 < *r* < –0.2	–0.7 < *r* < –0.2	–0.7 < *r* < –0.2	–0.7 < *r* < –0.2
Self-compassion	0.2 < *r* < 0.7	0.2 < *r* < 0.7	0.2 < *r* < 0.7	0.2 < *r* < 0.7	0.2 < *r* < 0.7
Depression		–0.7 < *r* < –0.2	–0.7 < *r* < –0.2	–0.7 < *r* < –0.2	–0.7 < *r* < –0.2
Anxiety		–0.7 < *r* < –0.2	–0.7 < *r* < –0.2	–0.7 < *r* < –0.2	–0.7 < *r* < –0.2

## Methods

### Participants

A cross-sectional survey was conducted among participants who registered with an online survey company, Rakuten Insight, Inc., in Japan, in February 2019. To include Japanese adults of a wide range of ages and genders, 14 blocks were set up, with seven categories by age (18–29, 30–39, 40–49, 50–59, 60–69, 70–79, and ≧ 80) and two by gender (male and female). Responses were collected so that each block had at least 50 respondents. As a result, 820 participants completed the self-reported questionnaires described in the “Measures” Section.

In addition, to examine the test-retest reliability, a longitudinal survey was conducted to measure the FFMQ twice with an interval of 4 weeks from November to December 2019. We aimed to obtain data from approximately 200 individuals and set 14 blocks with seven categories by age (18–29, 30–39, 40–49, 50–59, 60–69, 70–79, and ≧ 80) and two by gender (male and female), as in the above cross-sectional survey. Each block included at least 14 participants in order to obtain data from approximately 200 individuals (because 14 respondents × 14 blocks = 196). As a result, 267 responses were obtained in the first round of the longitudinal survey. Of these, 196 (14 randomly selected people in each block) people were asked to respond to the same questionnaire four weeks later, and 165 responses were obtained.

In both surveys, participants first read an explanation that this survey would be conducted anonymously and they would not be forced to respond. Subsequently, only those who agreed to participate in this study responded to the questionnaires. As a reward, respondents received points that they could redeem for goods within the system of the survey company. The Ethics Review Committee on Research with Human Subjects at Waseda University approved this study (application number: 2018-283). Written informed consent for participation was not required for this study in accordance with the institutional requirements.

### Measures

#### Dispositional Mindfulness

The Japanese version of the FFMQ (Sugiura et al., 2012; the original version, Baer et al., 2006) was used to assess the five aspects of mindfulness: observing (e.g., “I pay attention to sensations, such as the wind in my hair or sun on my face”), describing (e.g., “I’m good at finding the words to describe my feelings”), acting with awareness (e.g., “I do jobs or tasks automatically without being aware of what I’m doing” [a reverse item]) non-judging (e.g., “I tell myself I shouldn’t be feeling the way I’m feeling” [a reverse item]), and non-reactivity (e.g., “When I have distressing thoughts or images, I am able just to notice them without reacting”). This Japanese version of the FFMQ was translated from the original English version (Baer et al., 2006) by Sugiura et al. (2012) using a forward-backward method of translation. Items are rated using a 5-point Likert scale (1-never or very rarely true, 2-rarely true, 3-sometimes true, 4-often true, 5-very often or always true). A high total score indicates a high level of each aspect of mindfulness. Observing and non-reactivity are all comprised of positive items, acting with awareness and non-judging are all comprised of negative (reversed) items, and describing is comprised of both positive and negative items. The validity and reliability of this full version was confirmed by Sugiura et al. (2012). The full version with 39 items was used in this survey, and the short version (24-item and 15-item version) was analyzed by extracting the response data of the full version (see Table 2 for the item composition of the short versions).

**TABLE 2:** Descriptive statistics for FFMQ items and item composition of the short forms.

	Item		FFMQ						
Facet	No.		24	15	Mean	SD	Skewness	Kurtosis	% per response value
Observing	1				2.35	1.15	0.48	–0.63	29.02/27.78/26.55/12.04/4.61
	6			✓	2.25	1.13	0.71	–0.26	29.92/34.53/20.58/10.24/4.72
	11			✓	2.42	1.02	0.42	–0.37	19.12/37.57/28.35/12.15/2.81
	15		✓	✓	2.62	1.04	0.24	–0.42	15.07/30.37/36.11/13.95/4.50
	20		✓		2.74	0.97	0.05	–0.30	10.91/27.33/42.07/16.20/3.49
	26		✓		3.24	1.05	–0.11	–0.55	4.95/18.90/35.77/28.23/12.15
	31		✓		3.01	1.08	–0.05	–0.63	8.77/23.17/34.65/25.42/7.99
	36				2.91	0.95	0.09	–0.15	6.52/25.20/44.54/18.67/5.06
Describing	2		✓	✓	2.73	1.01	0.22	–0.35	10.57/31.27/37.23/15.97/4.95
	7		✓		2.86	1.04	0.10	–0.56	9.56/28.17/35.43/20.92/5.96
	12	*	✓		3.41	1.01	–0.29	–0.41	3.49/14.74/32.85/35.43/13.50
	16	*		✓	3.40	0.97	–0.28	–0.31	3.04/14.17/34.76/36.11/11.92
	22	*	✓		3.39	0.90	–0.32	–0.12	2.36/12.94/36.90/38.92/8.89
	27		✓	✓	2.82	0.95	0.12	–0.38	6.97/30.71/39.03/19.69/3.60
	32				2.64	0.98	0.36	–0.20	10.57/36.78/35.10/13.50/4.05
	37				2.80	0.98	0.23	–0.38	7.54/32.51/36.78/18.34/4.84
Acting with awareness	5	*			3.41	0.96	–0.23	–0.39	2.36/14.74/34.76/36.11/12.04
	8	*		✓	3.57	0.97	–0.44	–0.21	2.36/11.59/28.80/41.39/15.86
	13	*			3.43	1.02	–0.32	–0.45	3.37/15.19/30.93/36.45/14.06
	18	*	✓		3.57	0.92	–0.50	0.12	2.47/9.00/31.27/43.31/13.95
	23	*	✓		3.73	0.95	–0.48	–0.11	1.80/7.65/28.35/39.93/22.27
	28	*	✓		3.44	0.91	–0.27	–0.10	2.14/11.36/37.80/37.80/10.91
	34	*	✓	✓	3.63	0.89	–0.32	–0.15	1.24/8.21/32.96/41.28/16.31
	38	*	✓	✓	3.57	0.96	–0.33	–0.21	2.25/9.79/33.97/37.12/16.87
Non-judging	3	*			3.39	0.96	–0.20	–0.31	2.70/13.72/37.68/33.63/12.26
	10	*	✓	✓	3.41	0.97	–0.11	–0.44	2.14/14.06/38.13/31.61/14.06
	14	*		✓	3.67	1.00	–0.50	–0.11	3.04/7.87/30.26/36.56/22.27
	17	*	✓		2.90	0.99	0.06	–0.36	7.76/25.98/40.49/20.36/5.40
	25	*	✓		3.32	0.90	–0.03	–0.01	2.47/12.04/46.79/28.46/10.24
	30	*	✓	✓	3.48	0.89	–0.23	0.11	2.25/7.87/41.73/35.66/12.49
	35	*			3.14	0.95	–0.08	–0.24	4.27/19.01/42.52/27.22/6.97
	39	*	✓		3.34	0.96	–0.30	–0.07	4.27/12.15/39.71/33.52/10.35
Non-reactivity	4				2.70	0.93	0.24	–0.11	8.89/33.30/40.61/13.72/3.49
	9		✓		2.87	1.08	0.23	–0.64	8.66/31.83/31.50/20.13/7.87
	19		✓	✓	2.84	0.95	0.09	–0.36	7.20/29.25/39.93/19.80/3.82
	21				3.04	0.94	–0.06	–0.23	5.06/21.48/43.42/24.75/5.29
	24		✓		2.95	1.00	0.01	–0.50	7.20/25.87/37.35/23.96/5.62
	29		✓	✓	2.71	0.87	0.19	0.11	7.20/32.28/45.44/12.37/2.70
	33		✓	✓	2.69	0.90	0.10	0.00	9.11/30.48/45.33/12.49/2.59

(n = 889). “% per response value” indicates the percentage of participants responding in the first, second, third, fourth, and fifth category on the response scale. *Refers to a reverse item. All descriptive statistics were calculated after reverse scoring those items.

#### Mindful Attention and Awareness

The Japanese version of the MAAS (Fujino et al., 2015; the original version, Brown and Ryan, 2003) was used to assess the tendency of mindful attention and awareness of the present experience (e.g., “I tend to walk quickly to get where I’m going without paying attention to what I experience along the way” [a reverse item]). Items are rated on a 6-point Likert scale (1-almost never, 2-very infrequently, 3-somewhat infrequently, 4-somewhat frequently, 5-very frequently, 6-almost always). All items consist of reversed items only where they ask about the lack of mindful attention and awareness. Thus, a high total score after reverse scoring indicates a high level of mindful attention and awareness. The validity and reliability of this scale have been confirmed by Fujino et al. (2015).

#### Mind Wandering

The Japanese version of the Mind Wandering Questionnaire (MWQ; Kajimura and Nomura, 2016; the original version, Mrazek et al., 2013) was used to assess mind-wandering tendency (e.g., “I find myself listening with one ear, thinking about something else at the same time”). Items are rated using a 6-point Likert scale (1-almost never, 2-very infrequently, 3-somewhat infrequently; 4-somewhat frequently; 5-very frequently; 6-almost always). A high total score indicates a high level of mind-wandering tendency. The validity and reliability of this scale have been confirmed by Kajimura and Nomura (2016).

#### Interoceptive Awareness

The Japanese version of the Multidimensional Assessment of Interoceptive Awareness (MAIA-J; Shoji et al., 2018; the original version, Mehling et al., 2012) was used to assess interoceptive body awareness. The Japanese version of MAIA consists of the following six subscales: Noticing (e.g., “When I am tense I notice where the tension is located in my body”), Not-Distracting (e.g., “I distract myself from sensations of discomfort” [a reverse item]), Attention Regulation (e.g., “I can return awareness to my body if I am distracted”), Emotional Awareness (e.g., “I notice how my body changes when I feel happy/joyful.”), Body Listening (e.g., “I listen to my body to inform me about what to do”), and Trusting (e.g., “I feel my body is a safe place”). Not-Distracting consists of reversed items only, and the other subscales consist of positive items only. Items are rated on a 6-point Likert scale ranging from 0 (never) to 5 (always). The score for each subscale is calculated by averaging the item responses within the subscale. A high score on each subscale indicates a high level of interoceptive body awareness in daily life. The validity and reliability of this scale have been confirmed by Shoji et al. (2018).

#### Experiential Avoidance

The Japanese version of the Acceptance and Action Questionnaire - II (AAQ-II 7-item version; Shima et al., 2013; the original version, Bond et al., 2011) was used to assess experiential avoidance (e.g., “My painful experiences and memories make it difficult for me to live a life that I would value”). Items are rated using a 7-point Likert scale (1-never true, 2-very seldom true, 3-seldom true, 4-sometimes true, 5-frequently true, 6-almost always true, 7-always true). A high total score indicates a high level of experiential avoidance. The validity and reliability of this scale have been confirmed by Shima et al. (2013).

#### Cognitive Fusion

The Japanese version of the Cognitive Fusion Questionnaire (CFQ 7-item version; Shima et al., 2016; the original version, Gillanders et al., 2014) was used to assess cognitive fusion (e.g., “I get so caught up in my thoughts that I am unable to do the things that I most want to do”). Items are rated on a 7-point Likert scale (1-never true, 2-very seldom true, 3-seldom true, 4-sometimes true, 5-frequently true, 6-almost always true, 7-always true). A high total score indicates a high level of cognitive fusion. The validity and reliability of this scale have been confirmed by Shima et al. (2016).

#### Openness and Neuroticism (Big Five Personality)

The Japanese version of the Ten Item Personality Inventory (TIPI-J; Oshio et al., 2012; the original version, Gosling et al., 2003) was used to assess openness and neuroticism. We used the two subscales measuring openness (e.g., “I see myself as open to new experiences, complex”) and neuroticism (e.g., “I see myself as anxious, easily upset”), which comprise two items, respectively. Items are rated using a 7-point Likert scale (1-disagree strongly, 2-disagree moderately, 3-disagree a little, 4-neither agree nor disagree, 5-agree a little, 6-agree moderately, 7-agree strongly). A high total score on each scale indicates a high tendency of each personality dimension. The validity and reliability of this scale have been confirmed by Oshio et al. (2012).

#### Self-Compassion

The short form of the Japanese version of the Self-Compassion Scale (SCS; Arimitsu et al., 2016; the original version, Raes et al., 2011) was used to assess self-compassion. Although the SCS has been theorized to have a six-factor structure, confirmatory factor analysis of the higher two-factor model yielded sufficient fit indices comparable to the six-factor model of the short form of the Japanese version of the SCS (Arimitsu et al., 2016). Therefore, we used this scale based on the two-factor model with the positive and the negative factor for self-compassion. The positive factor contains items that directly refer to self-compassion, such as self-kindness (e.g., “When I’m going through a very hard time, I give myself the caring and tenderness I need”), and the negative factor contains items opposed to self-compassion, such as self-judgment (e.g., “I’m disapproving and judgmental about my own flaws and inadequacies”). Items are rated on a 5-point Likert scale ranging from 1 (almost never) to 5 (almost always). A higher total score of items on the positive factor indicates a higher level of self-compassion, while a higher total score of items on the negative factor indicates a lower level of self-compassion. The validity and reliability of this scale have been confirmed by Arimitsu et al. (2016).

#### Depression

The Japanese version of the Center for Epidemiologic Studies Depression Scale (CES-D; Shima et al., 1985; the original version, Radloff, 1977) was used to assess depressive symptoms. Items are rated on a 4-point Likert scale (0-rarely or none of the time, 1-some or little of the time, 2- moderately or much of the time, 3-most or almost all the time). A high total score indicates high levels of depressive symptoms. The validity and reliability of this scale have been confirmed by Shima et al. (1985).

#### Anxiety

The Japanese version of the State-Trait Anxiety Inventory (STAI; Hidano et al., 2000; the original version, Spielberger, 1983) was used to assess anxiety. We used the subscale measuring trait anxiety, although the inventory consists of two subscales (state and trait anxiety) with 20 items each. Items are rated using a 4-point Likert scale (1-almost never, 2-sometimes, 3-often, 4-almost always). A high total score indicates a high tendency of trait anxiety. The validity and reliability of this scale have been confirmed by Hidano et al. (2000).

### Data Cleaning

Surveys using online survey companies have revealed many satisficing or insufficient effort responses (Miura and Kobayashi, 2016) and have been analyzed after implementing some data cleaning procedures (e.g., Fujino et al., 2015; Erikawa and Yamada, 2018). As in a previous study using an online survey in Japan (Erikawa and Yamada, 2018), we excluded those who responded to the whole questionnaire too quickly (suspected fraudulent responses) and those who took too long (possible response interruptions). In the present study, because the mean and the distribution of response times were different between response devices (smartphone, PC, and the others), the data were first divided into responses on smartphones and on PCs and other devices (e.g., tablet). Then, for each group of devices, response times (the time from the start of answering the questionnaire to the end of answering it) were converted by natural logarithmic transformation, and we specified very short-time respondents as those with a response time less than –2 SD from the mean and very long-time respondents as those with a response time more than + 2 SD from the mean. For the smartphone sample, we excluded one very short-time respondent and five very long-time respondents, and for the PC and the others samples, we excluded 20 very short-time respondents and five very long-time respondents. Next, as in Fujino et al. (2015) who examined the validity of the Japanese version of the MAAS using an online survey company, we excluded 130 respondents who consistently gave the same answers (i.e., straight lining) other than the middle answer (e.g., 3 if it was a 5-point scale from 1 to 5) for a scale that included both positive and negative items in the subscale. Resultingly, 659 respondents were included in the analysis (Sample 1).

In the longitudinal survey to examine the test-retest reliability, the same data cleaning procedures as in the cross-sectional survey were applied to the first and second response data, respectively.

In the smartphone sample of the first response data, we excluded one very short-time respondent and four very long-time respondents, and in the PC and others samples, we excluded seven very short-time respondents and one very long-time respondent. Next, we excluded 18 respondents who had consistently given the same answers other than the middle answer in the FFMQ. We also excluded six respondents who had inconsistencies in their educational background with their second response data (e.g., a person who answered he graduated from a junior high school in his first response but answered he graduated from a university in his second response four weeks later). Therefore, 230 respondents were included in the analysis from the first response data (Sample 2). The demographic data for Sample 1 and Sample 2 (the first response) are shown in Table 3.

**TABLE 3:** Demographic data of the two samples.

	Sample 1 (*n* = 659)		Sample 2 (*n* = 230)	
	*n*	%	*n*	%
Male	311	47.2	112	48.7
Female	348	52.8	118	51.3
**Age (Male)**				
18–29	38	5.8	16	7.0
30–39	42	6.4	14	6.1
40–49	37	5.6	14	6.1
50–59	44	6.7	15	6.5
60–69	46	7.0	17	7.4
70–79	52	7.9	17	7.4
≧80	52	7.9	19	8.3
**Age (Female)**				
18–29	49	7.4	17	7.4
30–39	47	7.1	17	7.4
40–49	53	8.0	18	7.8
50–59	52	7.9	19	8.3
60–69	50	7.6	19	8.3
70–79	50	7.6	16	7.0
≧80	47	7.1	12	5.2
**Educational background**				
Elementary school	1	0.2	0	0.0
Junior high school	23	3.5	7	3.0
High school	191	29.0	68	29.6
Technical college	12	1.8	1	0.4
Vocational school	66	10.0	21	9.1
Junior college	59	9.0	22	9.6
University	276	41.9	95	41.3
Master’s Degree Program	21	3.2	8	3.5
Doctor’s Degree Program	8	1.2	4	1.7
Others	2	0.3	4	1.7
**Resident region**				
Hokkaido	30	4.6	16	7.0
Tohoku	28	4.2	9	3.9
Kanto	254	38.5	97	42.2
Chubu	80	12.1	32	13.9
Kinki	171	25.9	46	20.0
Chugoku	38	5.8	9	3.9
Shikoku	16	2.4	5	2.2
Kyushu	40	6.1	16	7.0
Okinawa	2	0.3	0	0.0
**Marital status/Family state**				
Single	151	22.9	55	23.9
Cohabiting	6	0.9	3	1.3
Married (Living together)	402	61.0	136	59.1
Married (Separated)	17	2.6	7	3.0
Divorced	42	6.4	13	5.7
Widowed	41	6.2	16	7.0

*Tohoku includes Aomori, Iwate, Miyagi, Akita, and Yamagata prefectures; Kanto includes Ibaraki, Tochigi, Gunma, Saitama, Chiba, Tokyo, and Kanagawa prefectures; Chubu includes Niigata, Toyama, Ishikawa, Fukui, Yamanashi, Nagano, Gifu, Shizuoka, Aichi prefectures; Kinki includes Mie, Shiga, Kyoto, Osaka, Hyogo, Nara, Wakayama prefectures; Chugoku includes Shimane, Tottori, Okayama, Hiroshima, and Yamaguchi prefectures; Shikoku includes Tokushima, Kagawa, Ehime, and Kochi prefectures; Kyushu includes Fukuoka, Saga, Nagasaki, Kumamoto, Oita, Miyazaki, and Kagoshima prefectures.*

In the smartphone sample of the second response data, we excluded the three very long-time respondents (overlapping with the one excluded in the first response data), and in the PC and others sample, we excluded the three very long-time respondents (overlapping with the one excluded in the first response data). After excluding those who were excluded from the first round of response data, we further excluded five respondents who showed a straightlining pattern of answering by selecting the same responses other than the middle one in the second round of the survey. As a result, 137 respondents were included in the analysis for the test-retest reliability.

### Analyses

In statistical analyses, to examine the factor structure, the following models were tested by confirmatory factor analyses: a five-factor correlation model or a hierarchical model assuming a second-order factor loaded on by the five factors, and models assuming or not assuming two uncorrelated method factors loaded on by the positive and negative items, respectively. These four models were examined for the full version, 24-item version, and 15-item version of the FFMQ, respectively. Additionally, the models assuming four factors, excluding the observing subscale, were tested in each model described above, because the four-factor model may be valid in a general population (e.g., Baer et al., 2006) that is not selected based on meditation experience. In all models, error terms were not allowed to correlate, and the variance of the factors was fixed to 1, which was identical to the setting in Bohlmeijer et al. (2011). In the original 39-item version given by Baer et al. (2006) and Sugiura et al. (2012), they performed parceling items. We did not do so here because we aimed to compare the models of the full version with ones of the 24-item and 15-item versions which have too few items to parcel. In other words, we conducted confirmatory factor analyses where each item loaded on factors in all models. These analyses were conducted using the combined data from the cross-sectional survey (sample 1) and the first response data from the longitudinal survey (sample 2), given that the response situations were the same, namely, both surveys were conducted by the same survey company, and the FFMQ was completed at the beginning of the survey.

Four fit indices were used to indicate the global fit of the models to the data: the comparative fit index (CFI), the root mean square error of approximation (RMSEA), the standardized root mean square residual (SRMR), and the chi-square model test. Rules of thumb for the cutoff values that indicate acceptable fits are as follows: CFI values ≥ 0.90 and 0.95 were considered indicative of acceptable and good model fit, respectively. RMSEA smaller than 0.06 and the SRMR smaller than 0.08 indicate a good fit (Hu and Bentler, 1999). The chi-square test is generally not recommended for assessing model fit due to its sensitivity to non-normality, large correlations between variables, large sample size, and variables with high proportions of unique variance (Kline, 2011). Therefore, following Gu et al. (2016), we reported the results of the chi-square tests along with other fit measures but did not use it as a primary measure of model fit.

Next, in order to estimate reliability, Cronbach’s alpha coefficient and McDonald’s omega were calculated for each subscale of each version of the FFMQ for the combined sample. Values of 0.7 or higher for both indices were considered to indicate sufficient reliability.

In addition, to examine test-retest reliability, the intraclass correlation (2, 1) was calculated for each subscale of each version of the FFMQ for 137 participants who responded to both the first and second surveys in the longitudinal study. Values below 0.4 were interpreted as “poor,” values between 0.40 and 0.59 as “fair,” values between 0.60 and 0.74 as “good,” and values above 0.75 as “excellent” (Cicchetti, 1994).

In order to examine whether the original and shortened versions of the FFMQ share the same information, Pearson’s correlation coefficients between the subscales of the original and shortened versions of the FFMQ were calculated for the combined sample. Values of 0.9 or higher were considered, as both versions shared the same information.

To examine construct validity, Pearson’s correlation coefficients between the original and shortened versions of the FFMQ and the other scales were calculated for sample 1, in which participants responded to all the questionnaires described above.

Only intraclass correlation was calculated using IBM SPSS Statistics 27. All the other analyses, including the calculation of descriptive statistics, were performed using a solver-on version of HAD17_202, which is a statistical software based on Microsoft Excel (Shimizu, 2016).

## Results

### Descriptive Characteristics

Table 2 shows the mean, standard deviation, kurtosis, skewness, and percentage per response value for each item of the FFMQ, for samples 1 and 2 combined. The descriptive characteristics for the subscales of the FFMQ and the other variables are presented in Table 4, including mean, standard deviation, kurtosis, and skewness. Acceptable levels of kurtosis and skewness (i.e., between –1 and +1; Oguchi et al., 2021) were observed for all variables, indicating a normal distribution.

**TABLE 4:** Descriptive statistics for the subscales of each version of the FFMQ and the other scales.

	Mean	SD	Skewness	Kurtosis		Mean	SD	Skewness	Kurtosis
			
	*n* = 889 (sample 1 + 2)					*n* = 659 (sample 1)			
FFMQ					MAAS	62.76	10.69	–0.03	0.39
Observing					MWQ	15.01	4.83	–0.16	–0.12
FFMQ-39	21.54	5.31	0.11	0.37	MAIA				
FFMQ-24	11.61	3.00	–0.01	0.14	Noticing	2.38	0.94	0.14	0.10
FFMQ-15	7.30	2.42	0.30	−0.10	Not-distracting	2.80	1.05	−0.02	−0.13
Describing					Attention regulation	2.57	0.92	0.02	0.37
FFMQ-39	24.05	5.33	0.19	0.70	Emotional awareness	2.54	1.15	−0.17	−0.09
FFMQ-24	15.21	3.50	0.00	0.64	Body listening	2.29	1.06	−0.06	0.04
FFMQ-15	8.95	2.13	0.07	0.63	Trusting	2.50	1.10	0.01	−0.04
Acting with awareness					AAQ-II	20.25	8.35	0.66	0.81
FFMQ-39	28.34	5.26	−0.23	0.28	CFQ	19.11	10.00	0.67	−0.02
FFMQ-24	17.94	3.33	−0.20	0.32	TIPI				
FFMQ-15	10.77	2.21	−0.24	0.32	Openness	7.91	2.26	0.08	0.29
Non-judging					Neuroticism	7.45	2.69	0.09	−0.32
FFMQ-39	26.65	5.13	−0.02	0.54	SCS				
FFMQ-24	16.45	3.42	−0.05	0.45	Positive	17.86	4.56	−0.10	0.50
FFMQ-15	10.57	2.25	−0.14	0.17	Negative	16.25	5.30	0.14	−0.13
Non-reactivity					CES-D	15.92	10.40	0.88	0.28
FFMQ-39	19.79	4.24	−0.18	0.64	STAI-T	44.10	11.84	0.30	−0.02
FFMQ-24	14.06	3.21	−0.15	0.47					
FFMQ-15	8.24	2.02	−0.09	0.54					

*FFMQ, Five Facet Mindfulness Questionnaire; MAAS, Mindful Attention Awareness Scale; MWQ, Mind Wandering Questionnaire; MAIA, Multidimensional Assessment of Interoceptive Awareness; AAQ-II, Acceptance and Action Questionnaire – II; CFQ, Cognitive Fusion Questionnaire 7 item-version; TIPI, Ten Item Personality Inventory; SCS, the short form of the Self-Compassion Scale; CES-D, The Center for Epidemiologic Studies Depression Scale; STAI-T, the Trait subscale of the State-Trait Anxiety Inventory.*

### Confirmatory Factor Analysis

Confirmatory factor analyses were conducted on the five subscales and the four subscales except for the observing subscale, assuming five-factor correlations vs. a second-order factor, assuming vs. not assuming uncorrelated method factors loaded on by the positive and negative items, in full version, 24-item version, and 15-item version of the FFMQ, respectively. The results of fit indices are shown in Table 5. The results of the chi-square tests were significant for all models (*p* < 0.001). Acceptable goodness-of-fit values were obtained for the five-factor model assuming method factors in 39-item and the 24-item versions in terms of RMSEA. Only the five-factor correlation model assuming method factors in the 24-item version was acceptable in terms of SRMR (0.075), while the CFI showed marginally less value (0.899) than the criterion > 0.90. For the four-factor model, the models assuming method factors in the 24-item version showed acceptable goodness of fit in terms of CFI and SRMR. In contrast, the models assuming methods factors in the 15-item version showed abnormal goodness of fit, probably suggesting that the model fits were quite poor. Since the 15-item version did not show sufficient goodness of fit in any of the models, we report the results of the 24-item version in subsequent analyses.

**TABLE 5:** CFA fit indices for the correlated or hierarchical models with or without uncorrelated method factors in each item-version of the FFMQ.

	χ^2^	df	CFI	RMSEA	SRMR
**FFMQ-39 (five factors)**					
Correlated model	4763.675	692	0.706	0.081	0.126
Hierarchical model	5338.599	697	0.665	0.087	0.153
Correlated model with method factors	2560.994	653	0.862	0.057	0.095
Hierarchical model with method factors	2593.076	658	0.860	0.058	0.097
**FFMQ-24 (five factors)**					
Correlated model	2006.199	242	0.738	0.091	0.117
Hierarchical model	2324.514	247	0.692	0.097	0.144
Correlated model with method factors	898.149	218	0.899	0.059	0.075
Hierarchical model with method factors	934.303	223	0.894	0.060	0.088
**FFMQ-15 (five factors)**					
Correlated model	766.994	80	0.780	0.098	0.106
Hierarchical model	1026.506	85	0.698	0.112	0.124
Correlated model with method factors^a^	13326.435	65	−3.253	0.479	397.488
Hierarchical model with method factors	13371.996	70	−3.266	0.462	402.481
**FFMQ-39 without observing (four factors)**					
Correlated model	3757.176	428	0.703	0.094	0.138
Hierarchical model	4118.861	430	0.671	0.098	0.154
Correlated model with method factors	1669.852	397	0.886	0.060	0.084
Hierarchical model with method factors	1684.729	399	0.885	0.060	0.081
**FFMQ-24 without observing (four factors)**					
Correlated model	1702.538	164	0.728	0.103	0.124
Hierarchical model	1730.846	166	0.723	0.103	0.126
Correlated model with method factors	614.611	144	0.917	0.061	0.068
Hierarchical model with method factors	646.444	146	0.911	0.062	0.071
**FFMQ-15 without observing (four factors)**					
Correlated model	644.155	48	0.759	0.118	0.118
Hierarchical model	699.730	50	0.737	0.121	0.104
Correlated model with method factors	19511.195	36	−6.889	0.780	17166.507
Hierarchical model with method factors^a^	19525.204	38	−6.894	0.760	17184.897

*(n = 889). ^a^All models except for the correlated model including observing, and the hierarchical model not including observing, with method factors in the FFMQ-15 were estimated using the maximum likelihood method with the maximum number of iterations set to 10000 and the convergence criterion set to 0.00001. The correlated model including observing and the hierarchical model not including observing with method factors in the FFMQ-15 could not be estimated and the calculation overflowed in the setting, so the convergence criterion was changed to 0.0001.*

Table 6 shows the standardized factor loadings for the five-factor correlation model assuming method factors in the 24-item version, which showed the best fit in the short versions with the five subscales. All factor loadings were significant (*p* < 0.01) except the factor loading from the non-reactivity factor to item 9. All graphical models including the standardized factor loadings are shown in Supplementary Figure 1.

**TABLE 6:** Standardized factor loadings of the five-factor correlation model with uncorrelated positive and negative method factors in the 24 item-version of the FFMQ.

Subscale	Item No.		Factor 1	Factor 2	Factor 3	Factor 4	Factor 5	Factor 6	Factor 7	Communality
Observing	15		0.54	0	0	0	0	0.31	0	0.39
	20		0.64	0	0	0	0	0.27	0	0.48
	26		0.33	0	0	0	0	0.41	0	0.28
	31		0.35	0	0	0	0	0.54	0	0.42
Describing	2		0	0.47	0	0	0	0.44	0	0.42
	7		0	0.50	0	0	0	0.55	0	0.55
	12	*	0	0.57	0	0	0	0	0.57	0.65
	22	*	0	0.52	0	0	0	0	0.62	0.66
	27		0	0.31	0	0	0	0.66	0	0.53
Acting with awareness	18	*	0	0	0.17	0	0	0	0.55	0.33
	23	*	0	0	0.42	0	0	0	0.42	0.35
	28	*	0	0	0.31	0	0	0	0.48	0.33
	34	*	0	0	0.58	0	0	0	0.44	0.52
	38	*	0	0	0.48	0	0	0	0.56	0.54
Non-judging	10	*	0	0	0	0.56	0	0	0.47	0.53
	17	*	0	0	0	0.37	0	0	0.30	0.23
	25	*	0	0	0	0.57	0	0	0.48	0.55
	30	*	0	0	0	0.31	0	0	0.54	0.39
	39	*	0	0	0	0.29	0	0	0.58	0.42
Non-reactivity	9		0	0	0	0	0.03	0.43	0	0.19
	19		0	0	0	0	0.16	0.60	0	0.39
	24		0	0	0	0	0.16	0.62	0	0.41
	29		0	0	0	0	0.31	0.53	0	0.38
	33		0	0	0	0	0.60	0.31	0	0.45

(n = 889). *Refers to a reversed item. We assumed factors 6 and 7 as positive and negative method factors, respectively.

### Reliability Coefficients (Cronbach’s α and McDonald’s ω)

Cronbach’s α and McDonald’s ω of each subscale in the original and the 24-item version of the FFMQ are shown in Table 7. All subscales in the full version showed values > 0.7 that met the criterion. Describing, acting with awareness, and non-judging in the 24-item version showed values (α = 0.755, 0.766, 0.774; ω = 0.726, 0.771, 0.777, respectively) that met the criterion, while observing and non-reactivity showed marginally less values (α = 0.698, 0.685; ω = 0.699, 0.688, respectively) than the criterion.

**TABLE 7:** Reliability indices for facets of each item-version of the FFMQ.

	Cronbach’s α	McDonald’s ω
	**FFMQ-39**	
Observing	0.785	0.783
Describing	0.832	0.808
Acting with awareness	0.847	0.844
Non-judging	0.827	0.777
Non-reactivity	0.751	0.752
	**FFMQ-24**	
Observing	0.698	0.699
Describing	0.755	0.726
Acting with awareness	0.766	0.771
Non-judging	0.774	0.777
Non-reactivity	0.685	0.688

*(n = 889).*

### Test-Retest Reliability

Table 8 shows the results of intraclass correlation coefficients (2, 1). Describing and acting with awareness in the full version showed excellent and good values [ICC (2, 1) = 0.773, 0.715, respectively], while the other subscale (observing, non-judging, and non-reactivity) showed fair values [ICC (2, 1) = 0.586, 0.595, 0.575, respectively]. Describing and acting with awareness in the 24-item version showed good values [ICC (2, 1) = 0.741, 0.609, respectively], while the other subscales (observing, non-judging, and non-reactivity) showed fair values [ICC (2, 1) = 0.541, 0.551, 0.593, respectively].

**TABLE 8:** Intraclass correlation coefficients of facets in each item-version of the FFMQ.

	ICC	95%CI	
		Lower	Upper
	**FFMQ-39**		
Observing	0.586	0.464	0.686
Describing	0.773	0.695	0.832
Acting with awareness	0.715	0.622	0.788
Non-judging	0.595	0.476	0.693
Non-reactivity	0.575	0.452	0.677
	**FFMQ-24**		
Observing	0.541	0.411	0.650
Describing	0.741	0.655	0.808
Acting with awareness	0.609	0.491	0.704
Non-judging	0.551	0.423	0.658
Non-reactivity	0.593	0.473	0.691

*(n = 137).*

### Correlations Between Subscales in the Original and the Short Form of the Five Facet Mindfulness Questionnaire

Pearson’s correlations between the subscales in the original and the 24-item versions are shown in Table 9. The correlations between a subscale of the full version and the corresponding subscale of the 24-item version were above the criterion of 0.9 for four subscales except for observing (the correlation between observing subscales in the full and 24-item version was 0.899 before being rounded off).

**TABLE 9:** Pearson’s correlations between FFMQ subscales in each version.

		Observing				Describing				Acting with awareness				Non-judging				Non-reactivity	
Subscale	Ver.	39		24		39		24		39		24		39		24		39	
Observing	24	0.90	**																
Describing	39	0.26	**	0.25	**														
	24	0.21	**	0.19	**	0.97	**												
Acting with awareness	39	–0.26	**	–0.22	**	0.36	**	0.37	**										
	24	–0.24	**	–0.20	**	0.32	**	0.34	**	0.93	**								
Non-judging	39	–0.49	**	–0.41	**	0.10	**	0.14	**	0.55	**	0.51	**						
	24	–0.46	**	–0.39	**	0.09	*	0.12	**	0.50	**	0.46	**	0.96	**				
Non-reactivity	39	0.42	**	0.42	**	0.36	**	0.33	**	–0.03		–0.08	*	–0.22	**	–0.23	**		
	24	0.39	**	0.40	**	0.32	**	0.28	**	–0.07	*	–0.12	**	–0.23	**	–0.25	**	0.96	**

(n = 889). **p < 0.01, *p < 0.05.

### Correlations With the Related Concepts

Pearson’s correlations between each subscale of each version of the FFMQ and other related concept scales are shown in Table 10. The differences in correlations between the full version and other scales, and the 24-item version and ones, were so small that they almost fell within the range of ±0.1.

**TABLE 10:** Pearson’s correlations between the subscales of each version of the FFMQ and the other scales.

	Observing				Describing				Acting with awareness				Non-judging				Non-reactivity			
	39		24		39		24		39		24		39		24		39		24	
MAAS	–0.09	*	–0.07		0.39	**	0.40	**	0.73	**	0.70	**	0.46	**	0.42	**	0.05		0.02	
MWQ	0.06		0.05		–0.33	**	–0.31	**	–0.61	**	–0.52	**	–0.37	**	–0.33	**	–0.15	**	–0.11	**
MAIA																				
Noticing	0.36	**	0.34	**	0.18	**	0.15	**	–0.10	*	–0.11	**	–0.30	**	–0.28	**	0.17	**	0.15	**
Not-distracting	–0.19	**	–0.15	**	0.14	**	0.16	**	0.28	**	0.29	**	0.26	**	0.27	**	–0.10	*	–0.10	**
Attention regulation	0.36	**	0.32	**	0.36	**	0.33	**	0.15	**	0.11	**	–0.14	**	–0.17	**	0.40	**	0.36	**
Emotional awareness	0.41	**	0.37	**	0.28	**	0.24	**	0.05		0.03		–0.18	**	–0.18	**	0.24	**	0.21	**
Body listening	0.34	**	0.28	**	0.28	**	0.24	**	0.11	**	0.06		–0.12	**	–0.13	**	0.22	**	0.19	**
Trusting	0.29	**	0.27	**	0.37	**	0.33	**	0.22	**	0.17	**	–0.02		–0.04		0.31	**	0.28	**
AAQ-II	0.12	**	0.07		–0.32	**	–0.33	**	–0.47	**	–0.41	**	–0.44	**	–0.39	**	–0.26	**	–0.24	**
CFQ	0.14	**	0.08	*	–0.28	**	–0.29	**	–0.49	**	–0.42	**	–0.46	**	–0.41	**	–0.25	**	–0.23	**
TIPI																				
Openness	0.13	**	0.16	**	0.30	**	0.28	**	0.09	*	0.07		0.05		0.04		0.10	*	0.09	*
Neuroticism	0.02		0.01		–0.35	**	–0.35	**	–0.41	**	–0.34	**	–0.33	**	–0.28	**	–0.35	**	–0.31	**
SCS																				
Positive	0.27	**	0.26	**	0.31	**	0.28	**	0.12	**	0.09	*	–0.02		–0.03		0.37	**	0.35	**
Negative	0.08		0.06		–0.29	**	–0.29	**	–0.45	**	–0.37	**	–0.46	**	–0.41	**	–0.25	**	–0.22	**
CES-D	0.08	*	0.02		–0.27	**	–0.28	**	–0.45	**	–0.41	**	–0.36	**	–0.31	**	–0.23	**	–0.22	**
STAI-T	–0.06		–0.08	*	–0.39	**	–0.38	**	–0.43	**	–0.35	**	–0.32	**	–0.26	**	–0.37	**	–0.34	**

(n = 659). **p < 0.01, *p < 0.05, MAAS, Mindful Attention Awareness Scale; MWQ, Mind Wandering Questionnaire; MAIA, Multidimensional Assessment of Interoceptive Awareness; AAQ-II, Acceptance and Action Questionnaire − II; CFQ, Cognitive Fusion Questionnaire 7 item-version; TIPI, Ten Item Personality Inventory; SCS, the short form of the Self-Compassion Scale; CES-D, The Center for Epidemiologic Studies Depression Scale; STAI-T, the Trait subscale of the State-Trait Anxiety Inventory. A high score of AAQ-II indicates a high level of experiential avoidance.

## Discussion

The FFMQ, which measures the five aspects of mindfulness (i.e., observing, describing, acting with awareness, non-judging, and non-reactivity), is an important tool in mindfulness research; however, the short form has not been developed in Japan. This study aimed to examine the factor structure, reliability, and validity of the 24-item (Bohlmeijer et al., 2011) and 15-item (Baer et al., 2012) versions of the FFMQ in Japan.

The five-factor model assuming method factors in the 24-item version was shown to have the highest goodness of fit. The slightly higher goodness of fit of the five-factor correlation model compared to the hierarchical model reveals the same trend as in the original Japanese version of the FFMQ (Sugiura et al., 2012) and the original 24-item version (Bohlmeijer et al., 2011). In addition, the fit improved to an acceptable level by assuming the method factors, suggesting that the tendency to be influenced by the positively or negatively worded expressions may have been observed in the present sample. This is inconsistent with the results of the original Japanese version of the FFMQ (Sugiura et al., 2012), in which acceptable goodness of fit was found without assuming method factors. This may be due to the fact that while Sugiura et al. (2012) surveyed students at several universities in Japan, the present study surveyed a wide range of age groups throughout Japan using an online survey company. It has been shown that respondents are more likely to not read the instructions carefully, that is, to respond with insufficient effort, in surveys conducted by online survey companies such as the present study, than in ones that target university students (Miura and Kobayashi, 2016). The setting of the survey in the present study may have produced responses strongly influenced by the wording without fully understanding the meaning of the items. The scores of each facet of the FFMQ through online survey companies in Japan might be interpreted as being influenced not only by each mindfulness skill, but also by the wording of either the positive or negative items.

In the five-factor model, assuming method factors in the 24-item version, the loadings generally lay in the predicted direction, but only one item (item 9) of non-reactivity failed to show a significant loading. It may be due to the fact that all items of non-reactivity except for item 9 began with the phrase “When I have distressing thoughts or images,” which enquires about non-reactivity to thoughts and images. In contrast, item 9 enquires about non-reactivity toward feelings, suggesting that subtle differences in item wording may have affected the results. In this study, we decided to conduct subsequent analyses without excluding item 9 to enable international comparisons in the future and to measure a whole range of the facet contents.

Regarding reliability, the full version showed adequate reliability coefficients, and the 24-item version showed marginally acceptable reliability coefficients in terms of Cronbach’s α and McDonald’s ω. As for the test-retest reliability, all versions of the FFMQ demonstrated values above the fair value. However, the present study showed slightly lower values than the results of previous studies examining test-retest reliability of the full version of the FFMQ (Veehof et al., 2011, Dutch version: ICCs 0.61–0.84, 2-week interval, *n* = 38; Zhu et al., 2021, Chinese version: ICCs 0.61–0.86, 3-week interval, *n* = 53). Longer intervals between the initial test and the retest and larger sample sizes have been shown to lead to lower test-retest reliability coefficients (Oshio, 2016). Therefore, our results may have been affected by the slightly longer interval (4-week interval) and larger sample size (*n* = 137). Furthermore, it is reasonable that the test-retest reliability of the shortened version is lower than that of the full version because it has been shown that test-retest reliability tends to decrease as the number of items decreases (Oshio, 2016). Taken together, the relatively small test-retest reliability coefficients shown in this study are reasonable.

The correlation between the short version and the full version showed that the 24-item version shared almost 80% of the information with the full version (i.e., *r* > 0.90). In terms of the same criteria as in the previous study (Bohlmeijer et al., 2011), the 24-item version was shown to meet the same requirements as a shortened version of the FFMQ.

Regarding validity, correlations with other related concepts showed that the 24-item version had a close correlation pattern with the full version. There were some correlations that seemed to be influenced by the wording of the positive or negative items, but in general, the results supported the hypotheses as described in the Introduction. As predicted, acting with awareness in the shortened version of the FFMQ showed a strong positive correlation with the MAAS, which is the well-validated mindfulness scale (Qu et al., 2015). Similar to the correlation with the MAAS (Kajimura and Nomura, 2016), acting with awareness also showed a moderate negative correlation with the MWQ, which measures mind-wandering tendency, supporting our hypothesis. These results support the convergent validity of the shortened version of acting with awareness. In addition, describing showed small to moderate positive correlations with the subscales of the MAIA as predicted, but other subscales in the FFMQ showed different correlations. Observing and non-reactivity were positively correlated with the MAIA subscales except for not-distracting, while non-judging was negatively correlated with the MAIA subscales except for not-distracting. This result can be explained by the fact that observing and non-reactivity comprise only positive items, non-judging comprises only negative items, and only not-distracting in MAIA comprises solely negative items, and each subscale of FFMQ is partly explained by positive and negative item factors. In contrast, although acting with awareness is also a subscale consisting of negative items only, it is predictably positively correlated with some MAIA subscales, which partly supports the claim that awareness of interoceptive sensations is the basis of mindfulness skills. Variables related to adaptative functions, such as experiential avoidance and cognitive fusion, neuroticism of TIPI, a negative factor of SCS, depression, and anxiety, showed small to moderate correlations with facets other than observing in the FFMQ. Results similar to previous studies in the general sample (Baer et al., 2008; Sugiura et al., 2012) were replicated. In the Japanese general sample, it is possible that the skill of observing may not necessarily have adaptive functions, which is consistent with Sugiura et al. (2012). In addition, each facet of the FFMQ showed a small positive correlation with the openness of the big five personalities, which is similar to mindfulness in terms of open attitude but does not include the awareness component, supporting discriminant validity. Furthermore, facets except for non-judging of the FFMQ and the positive factor of self-compassion showed small positive correlations, supporting the argument that mindfulness and self-compassion are closely related in mindfulness training (Feldman and Kuyken, 2011). These results are generally supportive of the construct validity of the shortened versions of the FFMQ.

### Limitations

First, although it has been known that the functioning of the observing facet in the FFMQ may depend on the experience of mindfulness meditation (Baer et al., 2008), we did not enquire of meditation experience in this study. It was thought that few people practiced mindfulness meditation on a daily basis, given the prevalence of mindfulness in Japan in 2019 when the survey was conducted. However, we should have enquired of the same to consider the influence of meditation experience. In contrast, the result that only observing was not adaptively associated with variables related to mental health, such as depression, was replicated in the present study. Thus it is reasonable to view most of our sample as consisting of people without meditation experience.

Second, several subscales, including the full version of the FFMQ, showed not-so-high test-retest reliability. Given that the FFMQ will be used to examine time-series changes for practical settings, it may be necessary to reconsider the item composition or the content to ensure high test-retest reliability.

Third, our validation was only done in the general population, although Bohlmeijer et al. (2011) validated the 24-item version of the FFMQ in clinical populations. It is necessary to examine the validity and reliability of the Japanese version in clinical populations, such as those with depression and anxiety, which are often targeted by mindfulness interventions. In addition, the measurement equivalence in clinical and healthy populations, as well as in different age and gender groups, should be examined. Additionally, it is necessary to examine the sensitivity of the short form of the FFMQ to standard mindfulness interventions in the future.

## Conclusion

The 24-item version of the FFMQ was highly correlated with the original 39-item version and showed a similar factor structure and acceptable reliability and validity. However, the 15-item version did not show a reasonable factor structure. For valid measurement, the 24-item version (Supplementary Tables 1, 2) is relatively recommended. However, the reliability and validity of both versions in clinical groups or settings, and their sensitivity to intervention are unknown and need to be examined in the future.

## Data Availability Statement

The raw data supporting the conclusions of this article will be made available by the authors, without undue reservation.

## Ethics Statement

The studies involving human participants were reviewed and approved by Ethics Review Committee on Research with Human Subjects at the Waseda University. Written informed consent for participation was not required for this study in accordance with the national legislation and the institutional requirements.

## Author Contributions

TT designed and conducted the survey, undertook the statistical analyses, and wrote the draft manuscript. JS and MS supported the statistical analyses. MF and HK provided important suggestions regarding the analyses and the discussion of the results. All authors contributed to the article and approved the submitted version.

## Conflict of Interest

The authors declare that the research was conducted in the absence of any commercial or financial relationships that could be construed as a potential conflict of interest.

## Publisher’s Note

All claims expressed in this article are solely those of the authors and do not necessarily represent those of their affiliated organizations, or those of the publisher, the editors and the reviewers. Any product that may be evaluated in this article, or claim that may be made by its manufacturer, is not guaranteed or endorsed by the publisher.
