# Supplementary material for: The Validity and Reliability of the Short Form of the Five Facet Mindfulness Questionnaire in Japan
Source: Front Psychol. 2022 Apr 14;13:833381. doi: 10.3389/fpsyg.2022.833381 (PMC9046677; doi:10.3389/fpsyg.2022.833381)
Supplement: Supplementary file 1 [file Table_1.docx]

Supplementary Material

# Supplementary Tables

| **Supplementary Table 1** \| The items of the 24 item-version of the Five Facet Mindfulness Questionnaire | | | |
| --- | --- | --- | --- |
| Facet | Item No. |  | Item content |
| Observing | 15 |  | I pay attention to sensations, such as the wind in my hair or sun on my face.  髪に吹く風や、顔に当たる日光などの感覚に注意を向ける。 |
|  | 20 |  | I pay attention to sounds, such as clocks ticking, birds chirping, or cars passing.  時計が時を刻む音、鳥がさえずる声、車が通る音などの音に注意を向ける。 |
|  | 26 |  | I notice the smells and aromas of things.  物事の匂いや香りに気づく。 |
|  | 31 |  | I notice visual elements in art or nature, such as colors, shapes, textures, or patterns of light and shadow. 芸術や自然をみるとき、色、形、質感、光と影のパターンなどの視覚要素に注意を向ける。 |
| Describing | 2 |  | I’m good at finding words to describe my feelings.  自分の感情を表現する言葉を見つけるのが得意である。 |
|  | 7 |  | I can easily put my beliefs, opinions, and expectations into words.  私は、簡単に自分の信念、意見、期待を言葉にできる。 |
|  | 12 | * | It’s hard for me to find the words to describe what I’m thinking.  私にとって、自分が考えていることを表現する言葉を見つけるのは難しい。 |
|  | 22 | * | When I have a sensation in my body, it’s difficult for me to describe it because I can’t find the right words.  自分の身体に何かを感じた時、ぴったりとした言葉を見つけることができないために、それを表現するのが難しい。 |
|  | 27 |  | Even when I’m feeling terribly upset, I can find a way to put it into words.  ひどく混乱した時でさえ、何とかそれを言葉で表現できる。 |
| Acting with  awareness | 18 | * | I find it difficult to stay focused on what’s happening in the present.  目の前で起きていることに集中し続けるのが難しいと感じる。 |
|  | 23 | * | It seems I am “running on automatic” without much awareness of what I’m doing.  自分がしていることをあまり意識せずに「自動操縦」で動いているみたいである。 |
|  | 28 | * | I rush through activities without being really attentive to them.  十分に注意を払わずに、性急に物事をすすめる。 |
|  | 34 | * | I do jobs or tasks automatically without being aware of what I’m doing.  自分がしていることに注意を払わずに自動的に仕事をしている。 |
|  | 38 | * | I find myself doing things without paying attention.  気がつくと、注意を払わずに何かをしている。 |
| Nonjudging | 10 | * | I tell myself I shouldn’t be feeling the way I’m feeling.  自分の感じ方に対して、そんなふうに感じるべきではないと自分に言い聞かせる。 |
|  | 17 | * | I make judgments about whether my thoughts are good or bad.  自分の考えが良いか悪いか判断する。 |
|  | 25 | * | I tell myself that I shouldn’t be thinking the way I’m thinking. 自分の考え方に対して、そんなふうに考えるべきではないと自分に言い聞かせる。 |
|  | 30 | * | I think some of my emotions are bad or inappropriate and I shouldn’t feel them.  自分の感情のいくつかは不適当または不適切であり、それらを感じるべきではないと思う。 |
|  | 39 | * | I disapprove of myself when I have irrational ideas.  不合理な考えをいだいた時、自分に不満をいだく。 |
| Nonreactivity | 9 |  | I watch my feelings without getting lost in them. 感情を見守っていても、その中に迷い込むことはない。 |
|  | 19 |  | When I have distressing thoughts or images, I “step back” and am aware of the thought or image without getting taken over by it.  つらい考えやイメージが浮かんだとき、大抵それに心を占領されることなく、一歩下がってそれらを意識しておく。 |
|  | 24 |  | When I have distressing thoughts or images, I feel calm soon after.  つらい考えやイメージが浮かんだとき、大抵じきに気持ちが落ち着く。 |
|  | 29 |  | When I have distressing thoughts or images, I am able just to notice them without reacting.  つらい考えやイメージが浮かんだとき、大抵何とかしようとせずただそれらを見つめることができる。 |
|  | 33 |  | When I have distressing thoughts or images, I just notice them and let them go.  つらい考えやイメージが浮かんだとき、大抵それらに気づくだけで放っておく。 |
| * Refers to a reverse item. The item numbers correspond to ones of the original FFMQ items. | | | |

Supplementary Table 2 | The short form of the Five Facet Mindfulness Questionnaire in Japanese

| ●以下の質問は普段のあなたにどの程度あてはまるでしょうか．あてはまる数字を一つ〇でかこんでください．  1：まったくあてはまらない（あるいは非常にまれにしかあてはまらない） 2：めったにあてはまらない 3：たまにあてはまる 4：しばしばあてはまる 5：いつもあてはまる（非常にしばしばあてはまる） | | | まったくあてはまらない | めったにあてはまらない | たまにあてはまる | しばしばあてはまる | いつもあてはまる |
| --- | --- | --- | --- | --- | --- | --- | --- |
| 1. | (2) | 自分の感情を表現する言葉を見つけるのが得意である。 | 1 | 2 | 3 | 4 | 5 |
| 2. | (7) | 私は、簡単に自分の信念、意見、期待を言葉にできる。 | 1 | 2 | 3 | 4 | 5 |
| 3. | (9) | 感情を見守っていても、その中に迷い込むことはない。 | 1 | 2 | 3 | 4 | 5 |
| 4. | (10) | 自分の感じ方に対して、そんなふうに感じるべきではないと自分に言い聞かせる。 | 1 | 2 | 3 | 4 | 5 |
| 5. | (12) | 私にとって、自分が考えていることを表現する言葉を見つけるのは難しい。 | 1 | 2 | 3 | 4 | 5 |
| 6. | (15) | 髪に吹く風や、顔に当たる日光などの感覚に注意を向ける。 | 1 | 2 | 3 | 4 | 5 |
| 7. | (17) | 自分の考えが良いか悪いか判断する。 | 1 | 2 | 3 | 4 | 5 |
| 8. | (18) | 目の前で起きていることに集中し続けるのが難しいと感じる。 | 1 | 2 | 3 | 4 | 5 |
| 9. | (19) | つらい考えやイメージが浮かんだとき、大抵それに心を占領されることなく、一歩下がってそれらを意識しておく。 | 1 | 2 | 3 | 4 | 5 |
| 10. | (20) | 時計が時を刻む音、鳥がさえずる声、車が通る音などの音に注意を向ける。 | 1 | 2 | 3 | 4 | 5 |
| 11. | (22) | 自分の身体に何かを感じた時、ぴったりとした言葉を見つけることができないために、それを表現するのが難しい。 | 1 | 2 | 3 | 4 | 5 |
| 12. | (23) | 自分がしていることをあまり意識せずに「自動操縦」で動いているみたいである。 | 1 | 2 | 3 | 4 | 5 |
| 13. | (24) | つらい考えやイメージが浮かんだとき、大抵じきに気持ちが落ち着く。 | 1 | 2 | 3 | 4 | 5 |
| 14. | (25) | 自分の考え方に対して、そんなふうに考えるべきではないと自分に言い聞かせる。 | 1 | 2 | 3 | 4 | 5 |
| 15. | (26) | 物事の匂いや香りに気づく。 | 1 | 2 | 3 | 4 | 5 |
| 16. | (27) | ひどく混乱した時でさえ、何とかそれを言葉で表現できる。 | 1 | 2 | 3 | 4 | 5 |
| 17. | (28) | 十分に注意を払わずに、性急に物事をすすめる。 | 1 | 2 | 3 | 4 | 5 |
| 18. | (29) | つらい考えやイメージが浮かんだとき、大抵何とかしようとせずただそれらを見つめることができる。 | 1 | 2 | 3 | 4 | 5 |
| 19. | (30) | 自分の感情のいくつかは不適当または不適切であり、それらを感じるべきではないと思う。 | 1 | 2 | 3 | 4 | 5 |
| 20. | (31) | 芸術や自然をみるとき、色、形、質感、光と影のパターンなどの視覚要素に注意を向ける。 | 1 | 2 | 3 | 4 | 5 |
| 21. | (33) | つらい考えやイメージが浮かんだとき、大抵それらに気づくだけで放っておく。 | 1 | 2 | 3 | 4 | 5 |
| 22. | (34) | 自分がしていることに注意を払わずに自動的に仕事をしている。 | 1 | 2 | 3 | 4 | 5 |
| 23. | (38) | 気がつくと、注意を払わずに何かをしている。 | 1 | 2 | 3 | 4 | 5 |
| 24. | (39) | 不合理な考えをいだいた時、自分に不満をいだく。 | 1 | 2 | 3 | 4 | 5 |
| (The item numbers in parenthesis correspond to the original FFMQ items.) | | | | | | | |

# Supplementary Figure 1

Supplementary Figure 1 | Tested CFA models and the standardized factor loadings.

Note: The numbers in the names of the observed variables correspond to the item numbers in the original version of the FFMQ; obs = observing; des = describing; act = acting with awareness; nonj = nonjudging; nonr = nonreactivity; r = reverse-scored item.

FFMQ-39 (five factors) Correlated model

FFMQ-39 (five factors) Hierarchical model

FFMQ-39 (five factors) Correlated model with method factors

FFMQ-39 (five factors) Hierarchical model with method factors

FFMQ-24 (five factors) Correlated model

FFMQ-24 (five factors) Hierarchical model

FFMQ-24 (five factors) Correlated model with method factors

FFMQ-24 (five factors) Hierarchical model with method factors

FFMQ-15 (five factors) Correlated model

FFMQ-15 (five factors) Hierarchical model

FFMQ-15 (five factors) Correlated model with method factors

FFMQ-15 (five factors) Hierarchical model with method factors

FFMQ-39 without observing (four factors) Correlated model

FFMQ-39 without observing (four factors) Hierarchical model

FFMQ-39 without observing (four factors) Correlated model with method factors

FFMQ-39 without observing (four factors) Hierarchical model with method factors

FFMQ-24 without observing (four factors) Correlated model

FFMQ-24 without observing (four factors) Hierarchical model

FFMQ-24 without observing (four factors) Correlated model with method factors

FFMQ-24 without observing (four factors) Hierarchical model with method factors

FFMQ-15 without observing (four factors) Correlated model

FFMQ-15 without observing (four factors) Hierarchical model

FFMQ-15 without observing (four factors) Correlated model with method factors

FFMQ-15 without observing (four factors) Hierarchical model with method factors
